# Duodenal hematoma following endoscopic duodenal biopsy in an adult requiring arterial embolization and surgical evacuation: a case report and review of the literature

**DOI:** 10.3389/fgstr.2024.1409290

**Published:** 2024-08-01

**Authors:** Kelly L. Buchanan, Robert M. Wilechansky, Mythili P. Pathipati, Allan M. Goldstein, Daniel P. Ryan, Joseph C. Yarze

**Affiliations:** ^1^ Department of Medicine, Massachusetts General Hospital and Harvard Medical School, Boston, MA, United States; ^2^ Division of Gastroenterology, Massachusetts General Hospital and Harvard Medical School, Boston, MA, United States; ^3^ Division of Pediatric Surgery, Massachusetts General Hospital and Harvard Medical School, Boston, MA, United States

**Keywords:** duodenal hematoma, surgical evacuation, laparoscopic, endoscopic complications, arterial embolization

## Abstract

A 21-year-old man presented with severe abdominal pain four days after undergoing upper endoscopy with duodenal biopsies and was found to have an intramural duodenal hematoma. Symptoms progressed after attempts at diet advancement, and repeat imaging showed an enlarging hematoma with duodenal obstruction. The patient was managed with arterial embolization followed by laparoscopic surgical evacuation of the hematoma. This is the first report of an enlarging duodenal hematoma managed by this combination approach. While surgical interventions have previously been reserved for the most severe cases, we review the literature on minimally invasive approaches to manage this rare endoscopic complication.

## Introduction

Intramural duodenal hematoma is a rare complication of upper endoscopy. Information about its occurrence is limited to case reports, and data on incidence rates in adults are lacking. Duodenal hematoma after upper endoscopy is often related to tissue sampling, and the sequelae can be significant; patients may require several weeks of nutritional support due to duodenal obstruction (1–3). Early recognition is key to initiation of conservative management with nasogastric decompression and parenteral nutrition. Surgical decompression is considered in severe cases (4), and recent reports have raised the possibility of minimally invasive approaches to treatment (3, 5).

## Case report

**Figure 1:**
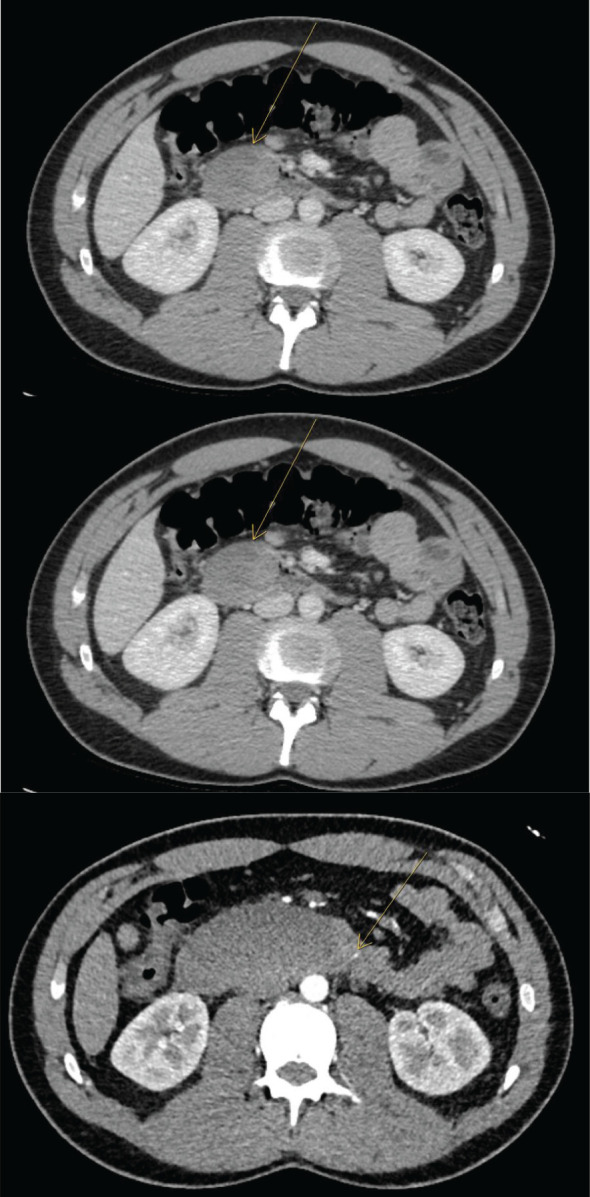
Progressive CT A/P images at day 1 and day 3 with expansion of duodenal hematoma and CT angio at day 5 with area of active extravasation.

A 21-year-old man with eosinophilic esophagitis was admitted with acute, severe abdominal pain. The pain initially began in the lower abdomen and progressed to involve the right upper quadrant and epigastrium. He had undergone upper endoscopy at another institution 4 days previously, at which time duodenal biopsies were performed to screen for celiac disease due to chronic upper gastrointestinal symptoms. The EGD was uneventful and there was not any report of excessive bleeding during the procedure. Pathology from these biopsies showed vascular congestion and erosion, negative for increased intraepithelial lymphocytes and negative for eosinophilic infiltration. On admission, his physical exam was notable for right upper quadrant and epigastric tenderness. Bloodwork revealed hypokalemia, and normal blood counts, liver enzymes and serum lipase. A computed tomography (CT) scan showed focal dilation of the second and third portions of the duodenum with luminal narrowing and an intramural duodenal hematoma extending from the second part of the duodenum (D2) to the ligament of Treitz, measuring 4.7 x 3.0 x 3.5 cm (**Figure 1**). Over the initial 24 hours of hospitalization, the patient developed nausea and bilious emesis with concern for intestinal obstruction. A repeat CT scan showed significant enlargement of the duodenal hematoma, measuring 3.9 x 8.3 x 4.5 cm (**Figure 1**). A nasogastric tube was placed with copious output. On the third day of hospitalization, due to progressive symptoms, he underwent CT angiography of the abdomen, which demonstrated a punctate focus of arterial phase enhancement consistent with active extravasation (**Figure 1**). Urgent gastroduodenal arterial embolization was performed. Nasogastric decompression was continued and parenteral feeding was instituted without clinical improvement. On day 7 of hospitalization, given continued lack of improvement with conservative treatment, surgical laparoscopic evacuation of the duodenal hematoma was performed after multidisciplinary discussion. During surgery, a bulging duodenal hematoma was visualized, submucosal blot clot was evacuated, and a drain was placed. Within hours of surgery, nasogastric tube output dropped precipitously, and the nasogastric tube was removed on post-operative day 2. Drain was removed on post-operative day 5. The diet was advanced successfully and the patient was discharged after 13 days of hospitalization.

## Discussion

Intramural duodenal hematoma is an exceedingly rare complication after endoscopic biopsy. Duodenal hematoma is more commonly caused by blunt abdominal trauma, particularly in children due to their thinner abdominal walls. In the post-endoscopy population, research to date has focused on its incidence in pediatric populations. In a single-center retrospective study of 26,905 pediatric patients, intramural duodenal hematoma after endoscopy had an incidence of approximately 1 in 2000 (1). Similar numbers in children have been reported at other institutions (2). The incidence rate of duodenal hematoma in adults has not been established, and may be even more rare. The first case report of duodenal hematoma after routine endoscopic biopsy in an adult was reported in 1989 (6), and since then, there have been only 10 additional reports (**Table 1** **).** Additional cases of duodenal hematoma have been reported after other endoscopic procedures [e.g. endoscopic retrograde cholangiopancreatography (7), ulcer therapy (8)], as well as in cases of blunt abdominal trauma, duodenal ulcers, and pancreatitis (9).

**Table 1:** Cases of duodenal hematoma after endoscopic biopsy in adults.

Treatment	Case report	Age	Sex	Risk factors	Symptom onset	Intervention	Time to intervention (days)	Time to improvement (days)
Conservative management	Zinelis, 1989	23	M	None	24 hours	NG tube, TPN	N/a	17
	Lipson, 1996 (1)	36	F	CML	6 hours	NG tube, TPN	N/a	11
	Worynski, 1998	23	M	CML, post-BMT	4 days	NG tube, TPN	N/a	Died on day 13
	Sgouros, 2002	32	M	Noonan’s syndrome	6 hours	NG tube, TPN	N/a	21
	Chen, 2006	39	M	None	Unknown	NG tube, TPN	N/a	7
	Hoenisch, 2011	21	F	None	24 hours	NG tube, TPN	N/a	19
	Henker, 2021	21	M	AML, post-BMT	24 hours	NG tube, TPN	N/a	21
Surgical management	Lipson, 1996 (2)	32	F	AML	16 hours	Surgical evacuation	16	Died on post-operative day 12
Minimally invasive management	Lloyd, 2004	18	F	None	1 day	Ultrasound-guided drainage	15	18
	Samra, 2018	28	M	None	A few hours	Endoscopic dilation	5	7

NG, Nasogastric; TPN, total parenteral nutrition; CML, chronic myelogenous leukemia; AML, acute myelogenous leukemia; BMT, bone marrow transplant. Time to intervention and time to improvement from reference of admission. N/a, Not Applicable.

Given the low incidence rate, the mechanism of and risk factors for duodenal hematoma development after endoscopy are poorly characterized. Proposed mechanisms include both macroscopic and microscopic factors. For example, the duodenum may be at increased risk for shear injury given that it is in a fixed retroperitoneal position adjacent to the lumbar spine (6). Further, it has a rich, highly vascularized submucosal plexus which can be prone to bleeding (2, 9). Procedure-related risk factors include method of sedation and positioning, while procedural experience has not been shown to be related to risk (1). Clinical risk factors for duodenal hematoma include coagulopathy, prior solid organ transplant, prior bone marrow transplant, and anticoagulation (1, 2, 6). Our patient had no known risk factors.

The natural history of duodenal hematoma has been established through case reports. Patients typically present within the first 72 hours following endoscopy (1); in this case, the patient presented at approximately 96 hours. Growth of the hematoma in this location can occlude the duodenal lumen causing proximal intestinal obstruction, resulting in vomiting. Compression and obstruction of the ampulla of Vater can lead to pancreatitis, and if left untreated, biliary obstruction may occur. In order to avoid these complications, we felt that surgical management would provide the most definitive solution given that surgical management has been the mainstay of treatment thus far. There are rare reports of more serious complications such as extraluminal rupture and hemoperitoneum (4).

The standard of care for duodenal hematoma after endoscopic biopsy is conservative management; of the 11 case reports in adults, 7 were managed conservatively with nasogastric decompression and parenteral nutrition (**Table 1**) (6, 10–14). In these patients, 3–4 weeks of parenteral nutrition was often required before clinical improvement. The other 4 patients received intervention: surgical evacuation (10), endoscopic dilation (3), or ultrasound-guided drainage (15). AXIOS stent is a possibility for management as well (16).

Here, we report the first case of a patient managed with arterial embolization to limit hematoma expansion followed by surgical evacuation to treat persistent duodenal obstruction. While conservative and surgical management have been the mainstays of treatment to date, advances in endoscopic and interventional radiology techniques have expanded treatment options. On review of duodenal hematoma reports across the spectrum of age and causes, 7 prior cases have been managed with minimally invasive techniques, including image-guided drainage (3, 15, 17, 18), endoscopic incision and drainage (7, 8), and endoscopy with dilation (3, 5). Arterial embolization represents a new approach for active bleeding (**Table 2**).

**Table 2:** Minimally invasive approaches to management of duodenal hematoma.

Case report	Age	Sex	Mechanism of injury	Intervention	Time to intervention (days)	Time to improvement (days)
Aizawa, 1990	52	M	Traumatic – fell striking abdomen	Ultrasound guided drainage followed by EGD with balloon catheter dilatation	0 to drainage, 7 to EGD	45
Lloyd, 2004	18	F	EGD with biopsies	Ultrasound-guided drainage	15	18
Gulotto, 2005	44	M	Traumatic – fell during seizure	CT-guided percutaneous drainage	15	21
Kwon, 2008	63	M	Post-treatment of duodenal ulcers with epinephrin and fibrin glue	Endoscopic incision and drainage	21	28
Pan, 2013	48	M	Post-ERCP with sphincterotomy	Endoscopic incision and drainage	1	5
Samra, 2018	28	M	EGD with biopsies	Dilation with endoscope	5	7
Alharbi, 2020	10	M	Traumatic – road traffic accident	Serial EGD with balloon dilation	5	7

EGD, Esophagogastroduodenoscopy; CT, computed tomography; ERCP, endoscopic retrograde cholangiopancreatography. Time to intervention and time to improvement from reference of admission.

In this case, we attempted conservative manage at first but the patient’s symptoms became worse each day. He initially did not have obstructive symptoms and developed these the course of his admission and was having difficulty tolerating tube feeds with an NG tube. Ultimately multiple options were presented to the patient, but given the rapid expansion of his hematoma and ongoing severe symptoms, we felt that surgical intervention would provide the most definitive and expeditious improvement which was in line with the patient’s preferences as well.

In conclusion, we describe an unusual case of duodenal hematoma in a 21-year-old man occurring after routine endoscopic duodenal biopsies. The patient had no clear risk factors, he presented more than 72 hours after endoscopy, and the hematoma enlarged by more than three-fold in the first 24-hours of hospital observation. Given rapid progression of the hematoma and persistent duodenal obstruction, the patient was managed with a combination of arterial embolization which successfully prevented further expansion of the hematoma, and subsequent laparoscopic hematoma evacuation. Further study and use of minimally invasive approaches for duodenal hematoma management could help reduce potential complications and time to recovery.

## Data availability statement

The original contributions presented in the study are included in the article/supplementary material. Further inquiries can be directed to the corresponding author.

## Ethics statement

Written informed consent was obtained from the individual(s) for the publication of any potentially identifiable images or data included in this article.

## Author contributions

KB: Writing – original draft, Writing – review & editing. RW: Writing – review & editing. MP: Writing – review & editing. AG: Writing – review & editing. DR: Writing – review & editing. JY: Writing – review & editing.
